# Emotional intelligence training intervention among trainee teachers: a quasi-experimental study

**DOI:** 10.1186/s41155-018-0112-1

**Published:** 2018-12-29

**Authors:** Raquel Gilar-Corbi, Teresa Pozo-Rico, Maria Luisa Pertegal-Felices, Barbara Sanchez

**Affiliations:** Department of Developmental Psychology and Didactics, University of Alicante, Campus San Vicente del Raspeig, Ap, 99, E-03080 Alicante, Spain

**Keywords:** Emotional intelligence, Training intervention, Quasi-experimental study, Higher education, Academic achievement, Multi-level analyses

## Abstract

**Background:**

Emotional intelligence (EI) has often been linked to improvements in professional performance. Indeed, generic competencies related to EI have been included in university curricula. However, learning EI involves significant time and effort on the part of students, and this may hinder the acquisition of specific content for each degree. In this study, an intervention to develop EI in higher education students is described and evaluated.

**Methods:**

The intervention consisted of eight group sessions performed in a regular course aiming to increase EI. The sessions included strategies and training on perceiving and understanding one’s own emotions and others’ emotions, identifying and understanding the impact one’s own feelings in adopting decisions, expressing one’s own emotions and the stress experienced, and managing both one’s own emotions and emotions of others. Participants were 192 students studying for a Master of Primary Education degree. A quasi-experimental nonequivalent control group pretest-posttest design was adopted. The effectiveness of the intervention was evaluated using multi-level analyses.

**Results:**

The results showed a significant improvement in the EI of students in the experimental group compared with the control group.

**Conclusions:**

This research demonstrates that it is possible to develop EI in higher education students, without hindering the acquisition of specific content competencies and, therefore, without interfering with their academic performance and without overburdening students with work outside the classroom.

**Trial registration:**

The experiment has been registered in the Initial Deposit of the Spanish Center for Sociological Research (CIS). 7/6/2015. http://www.cis.es/cis/opencms/ES/index.html.

## Background

Since 1990, when Salovey and Mayer (1990) first introduced the concept of emotional intelligence (EI) in the scientific literature—these researchers describe EI as a set of skills that involve the ability to identify and monitor their own thoughts, as well as those of others, using them to steer thinking and acting—numerous studies on the topic have been conducted. The benefits of EI have been documented in many areas of life but most frequently in professional fields (Stys & Brown, 2004). The relationship between emotional intelligence and performance in business has been supported by Boyatzis (2006, 2008), Brotheridge and Lee (2008), Cooper (1997), Dreyfus (2008), Koman and Wolff (2008), and Murga and Ortego (2003). EI contributes to job performance by allowing people to cultivate positive relationships, work effectively in teams, and build social capital (Caruso & Salovey, 2004; Goleman, 1998). EI may also contribute to performance by allowing people to regulate their emotions, to cope with stress effectively, to perform well under pressure, and to adapt to organizational changes (Lopes et al., 2006).

Within the field of education, the relationship between EI and different variables has also been frequently documented. The relationship between EI and the effectiveness of teachers has been researched by various authors. Jennings and Greenberg (2009) and Sutton and Wheatley (2003) described the close relationship between the Emotional Intelligence of professors and their effectiveness and quality during teaching and learning processes in the classroom. These authors also documented the development of prosocial behaviour in students and demonstrated the important role that emotions play for teachers, teaching, and students. In the same vein, Di Fabio and Palazzeschi (2008) evaluated the relationship between EI and self-efficacy in a sample of Italian professors and concluded that teacher self-efficacy is best explained along the intrapersonal dimension. Chan (2006) stated that the enhancement of EI could help teachers combat burnout. Later, Chan (2008) studied the relationships between EI, self-efficacy, and coping skills in professors from Hong Kong and found that intrapersonal and interpersonal emotional intelligence significantly predict teachers’ coping strategies. Colomeischia and Colomeischia (2014) described the relationships between EI, self-efficacy, work mentality, and job satisfaction and highlighted their importance in the development of strategies to improve the quality of training programmes for professors.

Chinese teachers think that emotions are a fundamental part of their work, so several studies have been carried out in Chinese schools in order to analyse the effects of EI on teachers. Thus, Lee and Yin (2011) analysed the emotional responses of Chinese teachers to educational reforms in secondary education in China. The study identified three types of teachers, with different emotional profiles, who were able to properly manage curricular reform in the Chinese context. Subsequently, the same authors Yin and Lee (2012) explored the emotional rules that govern the work of Chinese teachers and showed that there were four rules that govern their feelings and emotional expressions: a commitment to teach with passion, hiding negative emotions, keeping negative emotions in check, and manipulating (managing) emotions to achieve teaching objectives.

Broad agreement on the importance of developing social and Emotional Intelligence is evident (Bar-On, 2000; Domitrovich, Corest, & Greenberg, 2007; Elias & Clabby, 1992; Elias et al., 1986; Greenberg, Kusche, Cook, & Quamma, 1995; Nelson & Low, 2003; Nelis, Quoidbach, Mikolajczak, & Hansenne, 2009; Palomera, Fernández-Berrocal, & Brackett, 2008), but developing this competency requires the participation of the entire educational community, from primary school to university, in the process. Institutions at all levels often encounter great challenges in this effort (Elias et al., 1997; Zins, Weissberg, Wang, & Walberg, 2004).

In 1999, the Joint Declaration of the European Higher Education Area (Ministers of Education of the European Union, 1999) outlined approaches to creating a shared European space for higher education, with emphasis on the importance of students’ acquisition of abilities, skills, competencies and values; subsequently, in 2003, the Tuning Educational Structures in Europe Project (González & Wagenaar, 2003) outlined the professional profiles that should result from learning and acquisition of desirable competencies related to each area of study. Both reports examined competencies related to emotional intelligence (EI), such as interpersonal skills and the ability to work in teams.

In European university curricula, a series of both specific (particular to the degree) and generic competencies, which seek to train students to practice the profession specific to each degree in accordance with the demands of businesses, are included. Studies that have been conducted with university students show that they do not possess the competencies required by businesses for successful job market integration; employers demand more competencies than those exhibited by graduates (Proyecto Reflex ANECA Agencia Nacional de Evaluación de la Calidad y Acreditación, 2007; Pertegal-Felices, Jimeno-Morenilla & Sánchez-Romero, 2011). With regard to students seeking educational degrees, studies show that they lack sufficient skill in controlling their emotions, working in teams, managing other people, and adapting to continuous changes (Gilar, Martinez-Ruiz & Castejon, 2007; Pertegal-Felices, Jimeno-Morenilla & Sánchez-Romero, 2011; Pertegal-Felices, Castejón-Costa, & Jimeno-Morenilla, 2014).

If we direct our attention toward students, among studies that analyse the emotional profile of university students, we highlight those that relate it to burnout syndrome (Extremera & Durán, 2007; Weinstein, 2011), the development of the emotional competencies required by businesses (Molero & Reina-Estévez, 2012; Pertegal-Felices, Castejón-Costa, & Jimeno-Morenilla, 2014), and academic performance (Parker, Summerfeldt, Hogan, & Majeski, 2004). Several studies have demonstrated the existence of important links between EI and academic performance (Caruso & Howe, 2015). Using academic grades as an outcome measure, Barchard (2001), Brackett and Mayer (2003), and Lam and Kirby (2002) found a moderately strong relationship between EI and academic performance.

Therefore, it seems necessary to integrate the development of these competencies into educational curricula, starting with the initial training of teachers, to achieve effective professional development (Palomera et al., 2008; Pertegal-Felices, Jimeno-Morenilla & Sánchez-Romero, 2011).

For improving competencies related to EI, two main types of interventions can be found: interventions that are separate from the teaching provided and developed in the form of a course, and interventions that are integrated within the university curriculum as part of the course of study.

Among interventions developed in the form of a course, we highlight those developed by Short, Kinman, and Baker (2010), Yilmaz (2009), Bond and Manser (2009), and Oberst, Gallifa, Farriols, and Vilaregut (2009). Short et al. (2010) created a course in which coaching experts advised psychology students to promote their well-being, especially in the more stressful stages of university, as well as in lecture and seminar classes. The results of this study showed that the intervention groups had lower levels of stress; however, the impact of this effect on academic performance was not studied. Yilmaz (2009) conducted a study in which specific training was provided to university students to improve their anger management skills. This EI course was demonstrated to be effective in significantly lowering the anger levels of the students who received the intervention. In both studies, the EI intervention programmes were aimed at improving specific traits in university students.

With regard to interventions integrated into the university curriculum as part of regular studies, Shek et al. (2012) developed a positive youth development course at the University of Hong Kong entitled “Tomorrow’s Leader”. This course was included in the curriculum, promoted students’ interpersonal skills, and proposed different systems for evaluation. Pool and Qualter (2012) performed an intervention for students from different disciplines at an English university, but in this case, the 11-week programme was offered as an elective course for students in their second or third years. The intervention was successful because it improved the overall level of EI and emotional self-efficacy (ESE) of students in the intervention group.

### Assessment of emotional intelligence

There are different EI assessment instruments that are associated with the two broad models describing emotionally intelligent people: one model is based on skills, and the other is the trait EI model, in which skills are combined with certain personality traits.

The skills model considers EI a set of cognitive skills for using and adaptively managing emotions (Mayer, Caruso, & Salovey, 1999; Mayer & Salovey, 1997; Mayer, Salovey, & Caruso, 2000; Salovey & Mayer, 1990). Based on this model of intelligence, different instruments evaluating EI have been produced; these include both self-report and ability-tracking instruments. The Trait Meta-Mood Scale-48 (TMMS-48), developed by the Mayer and Salovey research group, and the ANECA Agencia Nacional de Evaluación de la Calidad y Acreditación, 2007 Self-Report Inventory (SSRI) by Schutte et al. (1998) are both self-report measures with acceptable values for internal consistency, reliability, and validity. In contrast, the Multifactor EI Scale (MEIS) by Mayer et al. (1999) and its shortened and improved version, the Mayer-Salovey-Caruso EI Test (MSCEIT) by Mayer, Salovey, and Caruso (2001), measure EI ability using a practical approach.

Self-report measures base their reliability on the sincerity of the subject and on how the subject perceives his/her behaviour in different situations presented by the test; these instruments thus provide a subjective perception of emotional skills. Tests based on abilities evaluate EI using a series of objective and impersonal questions. Specifically, the MSCEIT (which evaluates the ability to perceive, use, understand, and regulate emotions) is based on everyday scenarios and measures how well people perform their tasks and resolve emotional problems.

Models of trait EI operationalize EI as a construct in which certain key personality traits needed to develop emotionally intelligent behaviours are included (Bar-On, 2000; Cherniss, 2000; Goleman, 2001). From these models, different instruments based on self-report have been created: the Bar-On Emotional Quotient Inventory (EQ-i) (Bar-On, 1997); the Trait EI Questionnaire (TEIQue), which is similar to the measure by Bar-On (Petrides & Furham, 2003); and the Emotional Competence Inventory (ECI) (Boyatzis, Goleman, & Rhee, 2000) are highlighted.

The present study examined whether an intervention to develop EI may also have a positive influence on the emotional skill levels of university students. Our initial hypothesis is that participation in the EI development programme can improve emotional skills. We introduced the programme in the Educational Psychology course in a Primary School Teaching degree programme. Our research objectives included improving students’ emotional abilities, as measured by EQ-i self-reporting test by Bar-On (1997) (based on trait EI model) and the MSCEIT measure of ability (based on the skills model) (Mayer et al., 2001), without affecting syllabus content or student academic performance.

## Methods

### Participants

The total sample was composed of 192 trainee teachers who were enrolled in a required Educational Psychology course. The course is a part of the Primary School Teaching degree programme curriculum. A total of 68.8% of the participants were female and 31.3% were male. Participants’ average age was 19.72 years (SD = 4.53). Class groups were randomly assigned to the experimental (93 students) or control (99 students) groups. However, it should be noted that students were already grouped naturally in each of the class groups.

### Measures

The following measures were utilized in this study:

*Emotional Quotient Inventory: Short (EQ-i:S)* (Bar-On, 2002): This test is a shortened version of the Bar-On Emotional Quotient Inventory (Bar-On, 1997), Spanish adapted by MHS, Toronto, Canada. It consists of 51 items with values on a 5-point Likert-type scale, and it evaluates five general factors of EI: intrapersonal intelligence, interpersonal intelligence, adaptation, stress management, and general mood, and a total EI score. The EQ-i:S has adequate internal reliability with a total omega coefficient of .94, calculated from the parameter estimates of a confirmatory factor analysis in the validation sample (Bar-On, 2002). In the present study, the EQ-i total EI score was used.

*Mayer-Salovey-Caruso EI Test (MSCEIT)* (Mayer et al., 2001), translated into Spanish by Extremera and Fernández-Berrocal (2002): This test is an instrument for measuring EI, developed using the theoretical model of Mayer and Salovey (1997), who defined EI based on four basic skills: (a) perceiving emotions, (b) facilitating thought, (c) understanding emotions, and (d) managing emotions. The MSCEIT has an adequate internal reliability with a total omega coefficient of .78 (Fiori et al., 2014). In the present study, the MSCEIT total EI score was used.

Academic performance: This was evaluated using an objective test about contents of Educational Psychology course, which consisted of 40 questions with four response options; only one response was considered correct, and errors were penalized. The formula for correction was as follows: “Score = correct answers − (errors/*n* − 1)”, where *n* = the number of response options.

### Procedure

First, the students participating in this study were properly informed of the research. This study was carried out in accordance with the recommendations of University of Alicante Ethics Committee*.* The protocol was approved by the University of Alicante Ethics Committee (Ref. UA2015-07-06). All subjects gave written informed *consent* in accordance with the Declaration of Helsinki.

Then, the EQ-i questionnaire and the MSCEIT was administered in-person to each class at the first session of the Psychology of Education course. These instruments were implemented on the entire sample (192 students).

In the following practice sessions, the control group continued with its conventional programme of learning (using a methodology based on analysis and discussion of texts, and the resolution of practical cases relating to the body of theoretical content specific to the subject), whereas the experimental group completed a combination of the conventional programme and EI programme exercises, which were transversely linked to practical components of the course (Appendix 1). The intervention sessions included strategies and training on: (a) perceiving and understanding one’s own emotions; (b) perceiving and understanding others’ emotions; (c) identifying and understanding the impact one’s own feelings is having on thoughts, decisions, behaviour and performance at studies in order to make decisions and adapt to the demands of the situation; (d) expressing one’s own emotions and controlling the mood; (e) controlling the stress experienced; (f) managing one’s own emotions; and (g) influencing the moods and emotions of others. The comparison process of teaching group control and experimental group have been described in the Appendix 2.

The teachers responsible for the implementation of the programme were the same in all groups and comprised a member of the research team and a teaching professor for the Psychology of Education course.

Once the semester ended, the questionnaires were again administered (EQ-i and MSCEIT) in both the control group and the experimental group.

Finally, academic performance was evaluated in the entire sample at the end of the first-semester teaching period.

### Design and data analysis

A nonequivalent control group pretest-posttest design was adopted (Campbell & Stanley, 1966, 2015). Due to participants are nested within classes, the effectiveness of the intervention was evaluated using multi-level analysis.

Changes in EQ-i total EI score, and MSCEIT total EI score were compared between experimental and control groups using independent *t* test for continuous variables. The effectiveness of the intervention was evaluated using multi-level analysis (generalized linear mixed modeling adjusted for baseline covariates). Covariates included were age and gender. The level of significance was set at *α* = 0.05, and the null hypothesis rejected when *p* ≤ 0.05.

Finally, a comparison of means was performed to analyse whether there were significant differences in the performance of the experimental and control groups.

To conduct all of these statistical analyses, the Statistical Package for the Social Sciences (SPSS, version 21.0) was used.

## Results

First, to analyse whether there were differences in EI levels in the two groups before the intervention, mean contrast for the independent samples was completed using independent *t* test (continuous variables) or chi-square test (categorical variables). The results showed that there were no significant differences in any of the measured variables between the two groups in the pre-test phase (MSCEIT total, EQ-i total, age, and gender).

Changes in EI levels for the experimental and control groups in posttest phase are shown in Table 1. The results showed that there were significant differences and a big effect size in both variables between the two groups in the post-test phase.

**Table 1:** Contrast of means. *T* test for independent samples. Post-test phase

	*F*	Sig.	*t*	df	Sig.	Mean	SD	95% CI		*d*
MSCEIT total score	117.72	.00	− 13.42	97.23	.00	− 543.85	40.50	− 624.23	− 463.47	1.93
EQ-i total score	9.53	.00	− 9.13	170.49	.00	− 3.33	.36	− 4.05	− 2.61	1.31

Table 2 shows the changes in EI levels between pretest phase and posttest phase for the whole sample, experimental and control groups. The mean increased in EQ-i score for the whole sample and experimental group, and these changes were significant. The mean decreased in EQ-I score for the control group; however, this change was not significant. The mean increased in MSCEIT score for the whole sample, control, and experimental group, and these changes were significant. All effect sizes were small.

**Table 2:** Paired sample *T* test

	Mean	SD	SE	95% CI		t	Df	Sig.	d
Total sample MSCEIT	− 298.67	404.79	29.52	− 356.91	− 240.43	− 10.11	187	.00	.07
Total sample EQi	− 1.17	3.30	.23	− 1.64	− .70	− 4.9	191	.00	.04
Control group MSCEIT	− 8.15	24.87	2.55	− 13.22	− 3.09	− 3.19	94	.00	.00
Control group EQi	.42	2.91	.29	− .15	1.00	1.44	98	.15	.01
Experimental group MSCEIT	− 595.44	395.29	40.98	− 676.85	− 514.03	− 14.52	92	.00	.13
Experimental group EQi	− 2.87	2.81	.29	− 3.45	− 2.29	−9.83	92	.00	.12

For multi-level analysis using generalized linear mixed modeling, classroom and student ID were selected as subject fields. EQ-i total post-test score was selected as target and linear model is selected in the Target Distribution and Relationship (Link) with the Linear Model group. The group (control or experimental), age, gender, and EQ-i Total pre-test score were selected to create the main effects. A random effect block was created for classroom. Additionally, this model was compared with a linear regression model. For that, the random effect was deleted. And the model summary views (Table 3) provide some statistical evidence that there were no significant differences between the two models. Detailed results for linear mixed model are presented below.

**Table 3:** Fit index for linear mixed model and linear regression model (EQ-i as target)

Fit index	Linear mixed model	Linear regression	Chi-square difference	Sig.
-2LL	877.196	879.121	1.925	.17
AICC	881.261	881.142	.119	.73
BIC	887.658	884.352	3.306	.06

*-2LL* − 2 log likelihood, *AICC* Akaike information criterion corrected, *BIC* Bayesian information criterion

Results of the test of fixed effects sown that the model, and all effects except age and gender, appear to be statistically significant (corrected model: *F* = 16.79, *p* = .001; type of intervention (control/experimental): *F* = 32.32, *p* = .001; age: *F* = .78, *p* = .38; gender: *F* = .79, *p* = .37; total EQ-i pre-test: *F* = 31.72, *p* = .001).

Table 4 shows the parameter estimates for the overall model and individual effects. The coefficients showed the relationship of each model parameter to EQ-i total post-test score. All other things being equal, we would expect the EQ-i total post-test score of a student in the control group to be − 3.16 points lower than a student in the experimental group.

**Table 4:** Fixed coefficients. Target: EQ-i total post-test

Model term	Coefficient	Std. Error	*t*	Sig.	95% CI	
Intercept	24.20	2.45	9.87	.001	19.36	29.04
Intervention type = 1	− 3.16	.56	− 5.68	.001	− 4.26	− 2.07
Gender = 1	− .33	.37	− .89	.37	− 1.06	.40
Age	.03	.04	.88	.38	− .04	.11
Pre-test Total EQ-i	.38	.07	5.63	.001	.24	.50

Results of analysing the covariance parameter estimates and related statistics for residual and random effects showed a single variance estimate for the residuals of 5.41 (SD = .56, *z* = 9.61, *p* = .001, 95% CI 4.42 to 6.64). Nevertheless, the variance estimates for the intercept of the random effect with *classroom* defining subjects were .23 and resulted as not significant (SD = .33, z = .69, *p* = .48, 95% CI from .01 to 3.82).

Results from the multi-level analysis using generalized linear mixed model analysis with MSCEIT total post-test as target are presented below. Additionally, this model was compared with a linear regression model. For that, the random effect was deleted. Based on -2LL and AICC, the linear mixed model with one random intercepts is preferred over the linear regression model because linear mixed model has smaller values (Table 5). Detailed results for linear mixed model are presented below.

**Table 5:** Fit index for linear mixed model and linear regression model (MSCEIT as target)

Fit index	Linear mixed model	Linear regression	Chi-square difference	Sig.
-2LL	2463.413	2470.190	6.777	< .01
AICC	2467.480	2474.212	6.741	< .01
BIC	2473.832	2475.399	1.567	.21

*-2LL* − 2 log likelihood, *AICC* Akaike information criterion corrected, *BIC* Bayesian information criterion

Results of the test of fixed effects sown that the model, and all effects except age and gender, appear to be statistically significant (corrected model: *F* = 75.57, *p* = .001; type of intervention: *F* = 65.60, *p* = .001; age: *F* = .03, *p* = .85; gender: *F* = .50, *p* = .48; MSCEIT total pre-test: *F* = 218.21, *p* = .001).

Table 6 shows the parameter estimates table for the overall model and individual effects. The coefficients showed the relationship of each model parameter to MSCEIT total post-test score. All other things being equal, we would expect the MSCEIT Total post-test score of a student in the control group to be − 534.89 points lower than a student in experimental group.

**Table 6:** Fixed coefficients. Target: MSCEIT total post-test

Model term	Coefficient	Std. Error	*t*	Sig.	95% CI	
Intercept	2688.75	163.70	16.30	.001	2345.77	2991.75
Intervention type = 1	− 534.89	66.04	− 8.10	.001	− 665.19	− 404.60
Gender = 1	− 21.27	30.02	− .71	.48	− 80.50	37.96
Age	.56	3.03	− .18	.85	− 6.54	5.42
Pre-test Total MSCEIT	.50	.03	14.77	.001	.43	.57

Results of analysing the covariance parameter estimates and related statistics for residual and random effects shown a single variance estimate for the residuals of 32,641.05 (SD = 3437.16, z = 9.49, *p* = .001, 95% CI from 26,554.06 to 40,123.36). Nevertheless, the variance estimates for the intercept of the random effect with *classroom* defining subjects was 4158.49 and resulted as not significant (SD = 5275.73, z = .78, *p* = .43, 95% CI from 345.97 to 49,983.33).

**Fig. 1:**
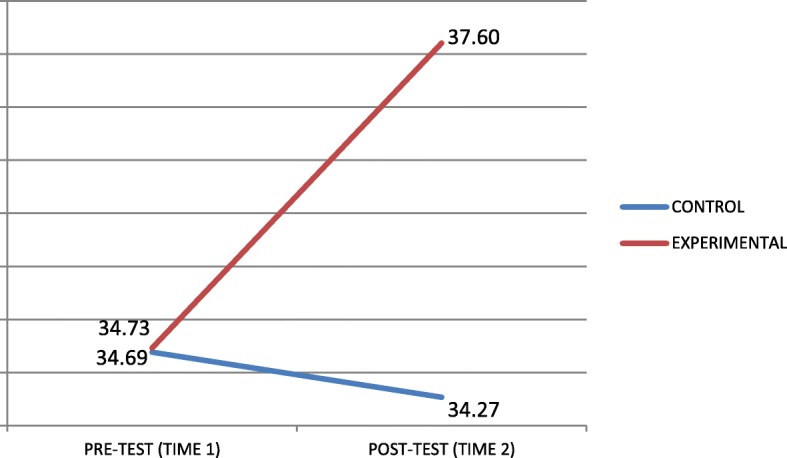
Graph of interactions for the EQ-I total score

**Fig. 2:**
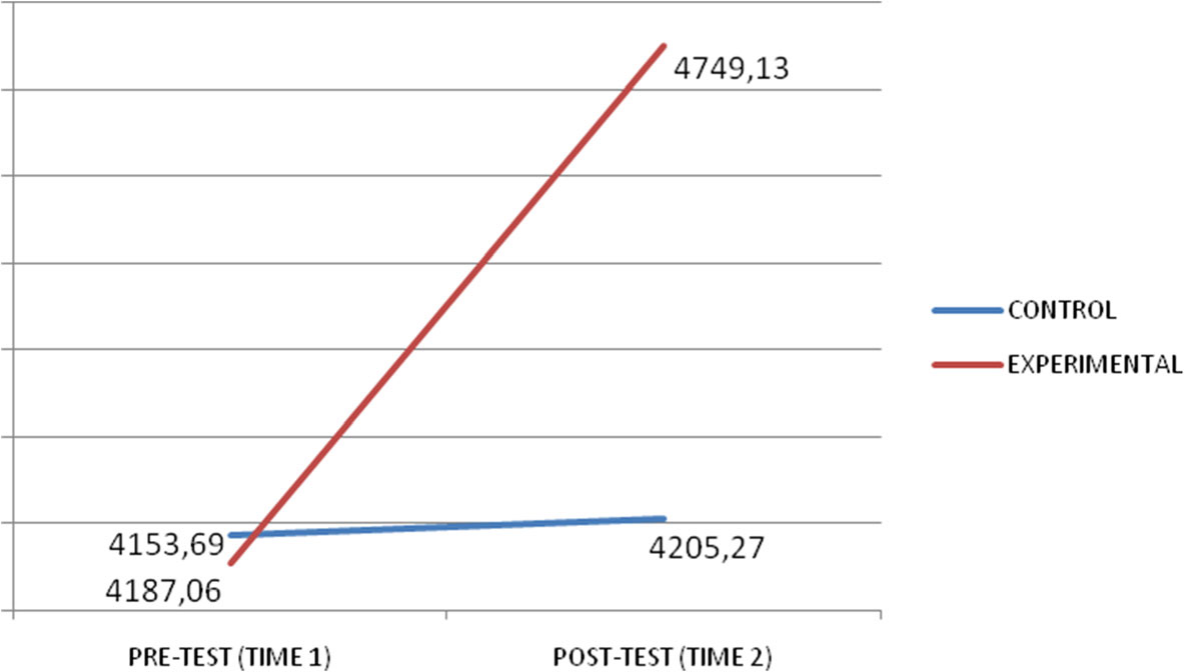
Graph of interactions for the MSCEIT total score

Figures 1 and 2 present interaction graphs that illustrate the directions of the differences. The experimental group’s total score in EQ-I and MSCEIT scales was greater once the intervention programme was completed.

Finally, we conducted an independent samples *t* test to determine if there were significant differences in the academic performance of the experimental and control groups. The results show significant differences in students’ performance and a big effect size, with higher scores for individuals in the experimental group (*t* = − 8.95, df = 170.88, *p* = .001, mean difference = − 1.05, 95% confidence interval − 1.29 to −.82, *d* = 1.30).

## Discussion

Numerous studies highlight the importance of EI in professional development (Boyatzis, 2006, 2008; Brotheridge and Lee, 2008; Cooper, 1997; Dreyfus, 2008; Koman & Wolff, 2008; Murga & Ortego, 2003). In the field of education, studies have also been published which demonstrate that teacher effectiveness and quality are associated with EI levels (Sutton & Wheatley, 2003; Di Fabio & Palazzeschi, 2008; Jennings & Greenberg, 2009). These and other studies also report other benefits of EI, such as improvements in trainee teachers’ prosocial behaviour, degree of job satisfaction, or the best ways to deal with strategies to improve educational programmes (Chan, 2008; Colomeischia & Colomeischia, 2014). Taking all this into account, this study presents a programme that is aimed at improving levels of EI in trainee teachers in order that trainee teachers may better develop their professional careers as future teachers.

The results of this study show that it is possible to develop emotional intelligence in university students within the curriculum, without interfering with students’ academic performance and without overburdening students with work outside the classroom.

It is important to say that in the school context, developing teachers’ basic ability to recognize emotions in other people helps to foster the specific competencies necessary for effectively resolving conflicts, such as an imminent classroom fights between students (Extremera & Fernández-Berrocal, 2004). In addition, the perception of emotions is a necessary prior skill for any emotional regulation strategy and is associated with the ability to respond empathetically to others (Mayer, Di Paolo, & Salovey, 1990).

As demonstrated by the results of performance in EQ-i, the students in the experimental group showed greater self-reported ability to control stress at the end of the semester, when they were facing pressure due to the approaching tests of academic performance. Farber (1991), more than a decade ago, highlighted the need to understand and address stress and burnout among teachers, so that American education could succeed; he proposed that the development of individual strategies to enable teachers to manage stress was a prerequisite for achieving such success in education. The student is the direct recipient of the consequences of stress in the teacher; stress management has beneficial preventative effects for teachers—it helps to moderate and prevent the negative effects of stress to which teachers are exposed on a daily basis; stress management also influences teachers’ work performance and the quality of their services in the classroom (Extremera & Fernández-Berrocal, 2004).

Additionally, we observed that the development of emotional intelligence in students did not hinder the acquisition of specific competencies.

Unlike other similar studies (Short et al., 2010; Yilmaz, 2009; Oberst et al., 2009), the programme to develop emotional intelligence was integrated into students’ regular syllabus and was not taught as a separate course. This decision risked overburdening students with extra work. These results can be considered very positive because in other completed studies (Bond & Manser, 2009), not only did the time dedicated to the programme involve a reduction in time dedicated to the specific content and, therefore, the students’ performance (in our study, EI time was shown to not have negative consequences on the students’ performance), but its completion also required greater effort, which students complained about due to the large amount of homework. Furthermore, the studies cited above do not assess the impact of their programmes on student achievement.

University students show a lack of emotional skills (Pertegal-Felices, Jimeno-Morenilla & Sánchez-Romero, 2011; Pertegal-Felices, Castejón-Costa, & Jimeno-Morenilla, 2014). Our results demonstrated that emotional intelligence can be trained in higher education and that the university environment presents the ideal climate in which to optimize the emotional management that strengthens multiple learning experiences.

Summarizing, the main objective of the present study was to examine whether an intervention to develop EI may have a positive influence on the emotional skill levels of university students. The results of this study show that it is possible to develop emotional intelligence in university students within the curriculum. This is a relevant result, since the emotional skills have proved necessary for a good personal, work, and social adjustment.

The personal and socio-emotional skills possessed by teachers will have a major influence on the way they teach and on the type of relations they establish in the classroom. This means that teachers must be competent in personal and emotional skills (Ria, Serve, Saury, Theureau, & Durand, 2003). However, this need for personal and emotional training is often not reflected in teacher training programmes (Dobbins, Higgins, Pierce, Tandy, & Tincani, 2010; Naeem et al., 2014; Sanchez-Ruiz, Mavroveli, & Poullis, 2013).

In a survey of 1281 Chinese schoolteachers, Yin, Lee, Zhang, and Jin (2013) found that the Emotional Intelligence of teachers, and the use of different emotional work strategies, have a significant impact on teacher satisfaction. This study demonstrated the beneficial role of EI on teacher’s wellbeing and emotional work, and the effectiveness of different emotional work strategies, and teacher trainings.

Weare and Gray (2003) recommend that socio-emotional skills should also be explicitly developed in institutions providing teacher training. This is based on the idea that it is impossible to teach a skill that has not been previously attained.

Existing studies show that great importance is attached to personal and socio-emotional skills in teachers’ professional work. Some studies (Jennings, 2011; Sutton & Wheatley, 2003) showed the close relation between teachers’ socio-emotional skills and their effectiveness and quality during teaching and learning processes in the classroom. The research shows that adequate levels of emotional intelligence help teachers to cope with the problems they face in the educational context more successfully (Berkovich & Eyal, 2015; Ju, Lan, Li, Feng, & You, 2015; Wurf & Croft-Piggin, 2015; Yin, 2015).

On the other hand, Bond and Manser (2009) conducted a study in which they modified courses from the first semester of technology degree programmes to integrate EI course concepts. The time dedicated to the EI course involved a reduction in the time dedicated to the remaining lessons and, therefore, decreased students’ academic performance. Despite the effort made, students complained about the large amount of homework required for this course compared to other courses, and of the five dimensions developed in the course, only the dimension of self-awareness was improved in the intervention group. The authors explained that this did not improve students’ overall level of EI due to the short duration of the course (15 weeks). In contrast, Oberst et al. (2009) proposed an intervention for psychology students that took the form of seminars directed at problem solving; these seminars were student-focused, with the objective of promoting emotional competencies and EI. They came to the conclusion that major changes in the university structure are needed to include this type of teaching and that, furthermore, it is necessary to devote a significant amount of time to it.

However, with the intervention that has been described in our study, the improvement achieved in the EI of the participants has been possible without interfering with students’ academic performance and without overburdening students with work outside the classroom.

Overall, the results of this study showed that the described intervention is effective for improving both self-reported EI and the ability for perceiving emotions, facilitating thought, understanding emotions, and managing emotions in order to solve emotional situations.

As a limitation of the study, we do not know if the success of the programme can be maintained in the long term, that is, we do not know whether the proposed training can strengthen and improve the initial levels of emotional intelligence acquired by the students. For this reason, we believe it would be desirable to undertake a longitudinal version of this study.

The sample on which this programme was implemented was also exclusively made up of Education Science students. Since the programme can be implemented in any university degree programme, we propose an evaluation of the effectiveness of its implementation in degrees connected with other areas of knowledge.

Possible future research could aim to increase both emotional intelligence and student performance with the development of programmes of the type described herein. Furthermore, doing so will provide scientific evidence concerning the usefulness of EI as a predictor of academic success. According to Zeidner, Roberts, and Matthews (2002), the studies that have been conducted to date are insufficient for verifying that students who possess higher levels of EI on some dimensions obtain higher academic scores. This lack of scientific evidence could be due to contradictory or inconclusive results, due to evaluation difficulty (Newsome, Day, & Catano, 2000); a lack of awareness of existing and adequate evaluation tools including those in the scientific, school, clinical, and organizational domains (Extremera, Fernández-Berrocal, Mestre, & Guil, 2004); methodological differences inherent in the majority of studies (Parker et al., 2004); and the organizational contexts of the centres and general educational systems (Extremera & Durán, 2007). Further research could aim to confirm the long-term effects of this EI programme on academic performance in other courses during the degree.

## Conclusions

The results of this study show that participants of the experimental group achieve a significant improvement in perceiving and understanding one’s own emotions; perceiving and understanding others’ emotions; identifying and understanding the impact one’s own feelings is having on thoughts, decisions, behaviour and performance at work in order to make decisions and adapt to the demands of the situation; expressing one’s own emotions and controlling the mood; controlling the stress experienced; managing one’s own emotions; and influencing the moods and emotions of others.

Overall, the results are promising as they indicated that it is possible to develop emotional intelligence in university students within the curriculum, without interfering with students’ academic performance and without overburdening students with work outside the classroom. The described intervention is effective for improving both self-reported EI and the ability for perceiving emotions, facilitating thought, understanding emotions, and managing emotions in order to solve emotional situations. This is a relevant outcome, since the emotional skills have proven necessary for a good personal, work, and social adjustment.

## Appendix 1

### Description of the content of the training EI intervention

#### Learning Content

The program timetable was eight weeks with a face-to-face session of 95 min each. The contents worked in each session were the following:

- 1st Session: Introduction. In this first session, objectives and methodology was exposed.
- 2nd Session: Intrapersonal EI and Self-Perception. In this second session the identification of own emotions is worked. It’s about learning the skill of perceiving and understanding one’s own emotions
- 3rd Session: Interpersonal EI. In this third session the identification of others’ emotions is worked. It’s about learning the skill of perceiving and understanding others’ emotions.
- 4th Session: Adaptability and Decision Making. In this third session the objective is work the capacity to identify and understand the impact one’s own feelings is having on thoughts, decisions, behaviour and performance at studies in order to make decisions and adapt to the demands of the situation. It’s about learning the skill of utilizing emotional information in decision-making
- 5th Session: General Mood and Self-Expression. In this fifth session expressing one’s own emotions and the skill of effectively controlling the mood is worked. It’s about learning the skill of expressing one’s own emotions and the skill of effectively controlling the mood across resolution of practical cases that require innovative ideas.
- 6th Session: Stress Management. In this session the skill of effectively controlling the stress experienced is worked. It’s about learning the skill of effectively controlling the stress experienced across a discussion activity about typical stressful situations in the work place.
- 7th Session: Emotional Understanding & Emotion Management. In this last session the skill of effectively managing one’s own emotions is worked and also the skill of influencing the moods and emotions of others is worked across an exhibition and discussion on how to apply these skills in the real working environment day by day.
- 8th Session: Feedback on session. This is a session to present the conclusions of the training

#### Methodology

- In each of the lessons, first, the objective of the work block to be considered is defined. Then, the class is divided into learning sections composed of groups of four to six students who are asked about relevant topics. Within each group, there is a leader who is selected by the group and who collects the main ideas that are discussed within the group during a period of approximately 10 or 15 min. The roles are interchanged (with all group members being leaders at some point), and the participants also rotate through the topics discussed in each learning section. Finally, the spokesperson states the findings, and if necessary, a new round begins with questions to be addressed in each of the created sections. Once these rounds have been completed, the spokespeople present the fundamental ideas provided by everyone who has participated during the different rounds. The ideas are added to the model using different techniques, such as conceptual maps.
- At the end, each student prepares an individual final conclusion. Finally, the teacher reviews the results of the session to prepare the next lesson based on the students’ pace of learning.

## Appendix 2

### C*omparison process of teaching group control and experimental group*

AIM: The subject educational psychology gives basic information about the main variables related to students and with a clear impact on learning. It also presents the explanatory theories of the acquisition process of abilities and school competencies, as a result of the learning process, as well as the interactions between interpersonal and contextual variables. This subject establishes the structure for students to understand their own students’ characteristics and the way of optimizing their learning. Control and Experimental Group develop the same theoretical and practical competences. However, the experimental group related the practical learning with IE skills.

**Table:**

	Theoretical Learning		Practical Learning	
	Control Group	Experimental Group	Control Group	Experimental Group
1st Session	Assume that working as a teacher means improving, updating and adapting to scientific, pedagogic, social and cultural change.		To understand the current situation of Educational Psychology as a discipline, its study goals and main context.	To understand the current situation of Educational Psychology as a discipline and IE Introduction.
2nd Session	Understand the characteristics and conditions in which school learning takes place and identify how this could affect the development of the students.		To understand concepts, methods and techniques related to the learning process in the educational context.	To understand concepts, methods and techniques to the learning process related to Intrapersonal EI and Self-Perception.
3rd Session	Organize teaching in the framework of the epistemological paradigms, showing one’s understanding of the learning aims of the areas of knowledge laid down in the primary education curriculum.		To analyze and synthesize the most significant explanatory models of school learning	To analyze and synthesize the most significant explanatory models of school learning across the developing of the Interpersonal EI skill.
4th Session	To act as a tutor, focusing on the students and parents of your group. All seeking understanding and cooperation with the families, bearing in mind different family contexts and lifestyles.		To organize and plan the educational situation, according to the students’ characteristics.	To organize and plan the educational situation across the developing of the Adaptability and Decision Making skills.
5th Session	Promote self-learning student’s skills, starting with the targets and content of each educational level, with positive expectations of student progress.		To apply the principles of knowledge acquisition to the design and development of teaching.	To apply the principles of knowledge acquisition across the developing of the General Mood and Self-Expression IE skills.
6th Session	Assess the social and environmental impact of actions taken in one’s field.		To resolve educational situations through the design of global learning environments in primary education.	To resolve educational situations through the design of global learning environments and developing Stress Management skill.
7th Session	Work in a team, collaborating and leading when necessary.		To develop the capacity of participation and negotiation in cooperative classroom work.	To develop the capacity of participation and negotiation in cooperative classroom work related with the develop of the Emotional Understanding & Emotion Management skills.
8th Session	Plan, organise and manage processes, information, problem-solving and projects. Possess initiative, entrepreneurial spirit and the ability to generate new ideas and actions.		To demonstrate the capacity for autonomous work.	To demonstrate the capacity for autonomous work and present the conclusions of the IE training

## Abbreviations

-2LL: − 2 log likelihood
AICC: Akaike information criterion corrected
BIC: Bayesian information criterion
CI: Confidence interval
*d*: Cohen’s *d*
Df: Degrees of freedom
ECI: Emotional Competence Inventory
EI: Emotional intelligence
EQ-i: Bar-On Emotional Quotient Inventory
EQ-i:S: Emotional Quotient Inventory: Short
*F*: F-test of the equality of two variances
MSCEIT: Mayer-Salovey-Caruso EI Test
*p*: *p* value
SD: Standard deviation
SE: Standard error
Sig: Statistical testing of significance
SPSS: Statistical Package for the Social Sciences
*t*: *t*-statistic
TEIQue: Trait Emotional Intelligence Questionnaire
*z*: *z* test

## Authors’ information

*Raquel Gilar-Corbí*. In 2002, she obtained her PhD in educational psychology. She is a lecturer at the Department of Developmental Psychology and Didactics at University of Alicante, Spain, since 2003. She currently is the director of the Department of Developmental Psychology and Didactics. Dr. Gilar has managed and participated in numerous research projects, both national and international. Her research interests are focused on intelligence, underachievement, high abilities, emotional intelligence, and teacher training.

*Teresa Pozo-Rico* received her PhD in 2011. She is an associate professor at the Department of Developmental Psychology and Didactics at University of Alicante and at the National Distance Education University (SPAIN). She works as advisor of teachers’ programmes and responsible for courses for Primary and Secondary School at the Education Establishment Training Teachers. Her lines of research revolve around the study of emotional intelligence and e-learning systems.

*María Luisa Pertegal-Felices* received the B.S. degree in Psychology and Pedagogy and the Ph.D. degree from the University of Alicante, Alicante, Spain, in 2011. She is currently an Assistant Lecturer with the Department of Developmental Psychology and Didactics, University of Alicante. She also has more than 10 years of child guidance experience at public schools. Her research interests include emotional intelligence applied to work performance, investigation of higher education, and the development of generic competencies.

*Bárbara Sánchez* received the B.S. degree in Psychology and Pedagogy from the University of Alicante, Spain, in 2004. Her research interests are focused on the study of personal intelligence and emotional intelligence applied to different educational stages in which she works as a teaching professional ranging from early education to university studies. She is an associate professor at the Department of Developmental Psychology and Didactics at University of Alicante since 2009. She has an experience of more than 18 years in teaching and 6 years as a member of the management team of a primary school.
